# Comprehensively Analyze the Prognosis Significance and Immune Implication of PTPRO in Lung Adenocarcinoma

**DOI:** 10.1155/2023/5248897

**Published:** 2023-02-09

**Authors:** Zhimin Lin, Jinjun Zhang, Biqiong Liu, Zhiqian Hong, Zengguang Chen, Xiaoyan Huang

**Affiliations:** ^1^Department of General Surgery, 900 Hospital of Joint Logistics Support Force, Fujian, Fuzhou, China; ^2^Fuzhou General Clinical Medical College of Fujian Medical University, Fujian, Fuzhou, China; ^3^The First Department of Surgery, Xianyou County General Hospital, Putian, Fujian, China; ^4^Department of Nephrology, Xianyou County General Hospital, Putian, Fujian, China; ^5^Longgang District Maternity & Child Healthcare Hospital of Shenzhen City, Shenzhen, Guangdong, China; ^6^Department of Orthopedics, Xiuyu District Hospital, Fujian, Putian, China; ^7^Department of Oncology, 900 Hospital of Joint Logistics Support Force, Fujian, Fuzhou, China

## Abstract

Immunotherapy for lung adenocarcinoma (LUAD) is considered to be a promising treatment option, but only a minority of patients benefit from it. Therefore, it is essential to clarify the regulation mechanism of the tumor immune microenvironment (TIM) of the LUAD. Receptor-type protein tyrosine phosphatase (PTPRO) has been shown to be a tumor suppressor in a variety of tumor; however, its role in LUAD has never been reported. In this study, we first found that PTPRO was lowly expressed in LUAD and positively correlated with patient prognosis. Next, we investigated the relationship between PTPRO and clinical characteristics, and the results showed that gender, age, T, and stage were closely related to the expression level of PTPRO. Moreover, we performed univariate and multivariate analyses, and the results revealed that PTPRO was a protective factor for LUAD. By constructing a nomogram based on the expression level of PTPRO and various clinical characteristics, it was proved that the nomogram has a good predictive capacity. Furthermore, we analyzed the coexpression network of PTPRO through multiple databases and performed GO and KEGG enrichment analyses. The results demonstrated that PTPRO was involved in the regulation of multiple immune pathways. In addition, we analyzed whether PTPRO expression of LUAD regulate immune cell infiltration and the results demonstrated that PTPRO was closely related to the infiltration of various immune cells. Finally, we predicted LUAD sensitivity to chemotherapeutics and response to immunotherapy by PTPRO expression levels. The results showed that PTPRO expression level affect the sensitivity of various chemotherapeutic drugs and may be involved in the efficacy of immunotherapy. These results we obtained suggested that PTPRO is closely related to the prognosis and TIM of LUAD, which may be a potential immunotherapeutic target for LUAD.

## 1. Introduction

Lung cancer (LC) is the second most common malignancy worldwide and one of the leading causes of cancer-related death currently [1]. LC is a heterogeneous malignancy, which is roughly divided into non-small-cell lung cancer (NSCLC) and small-cell lung cancer, and lung adenocarcinoma (LUAD) is the main pathological type of LC [2]. Currently, the traditional treatment options for LUAD mainly include surgery, chemotherapy, and radiation therapy [3]. However, the prognosis of LUAD is unsatisfactory, especially for advanced patients [4]. In the past two decades, with the further exploration of the mechanism of occurrence and development of LUAD, more treatment methods have been applied, such as targeted drugs and immunotherapy, which have significantly improved the prognosis of LUAD [5].

At present, a variety of immunotherapy methods have been developed, including vaccine therapy, chimeric antigen receptor (CAR) T cells, and immune checkpoint inhibitors (ICIs), including against cytotoxic T-lymphocyte-related antigen 4 (CTLA-4) antibodies, programming cell death 1 (PD-1), and programmatic cell death ligand 1 (PD-L1) [6]. The unique treatment effect of ICIs has gradually become a research hotspot in tumor treatment. Unfortunately, only a small percentage of patients benefit from immunotherapy [6]. Several studies have shown that the proportion of leukocytes infiltrating the tumor immune microenvironment (TIM) is closely related to the response to immunotherapy [7–10]. Therefore, elucidating the TIM regulation mechanism of LUAD is crucial for developing therapeutic strategies for LUAD.

Receptor-type protein tyrosine phosphatase (PTPRO) is a member of the PTP family and plays an important role in regulating human physiological and pathological processes [11, 12]. Based on previous studies, PTPRO has been shown to act as a tumor suppressor in the development of various tumors. The initial study found that overexpression of PTPRO inhibited the progression of lung cancer [13]. Another study revealed that PTPRO suppress tumor cell proliferation and promotes apoptosis by dephosphorylating signal transducer and activator of transcription 3 (STAT3) in liver cancer [14]. Not only that, PTPRO has also been found to be involved in regulating the TIM of various tumors in recent years. Gan and Zhang found that the expression level of PTPRO in human clear cell renal cell carcinoma is closely related to patient prognosis and immune infiltration [15]. Paradoxically, the expression level of PTPRO in pancreatic cancer is negatively correlated with patient prognosis and has the function of worsening the TIM [16]. However, there is no study of the relationship between PTPRO and immune function in LUAD.

In this study, we first analyzed the relationship between the PTPRO expression and prognosis in LUAD and further explored the correlation between PTPRO and clinical characteristics. Furthermore, the potential mechanism of PTPRO regulation of LUAD progression was explored by KEGG and GO analyses. In addition, we analyzed the correlation between PTPRO and immune cell infiltration status by multiple public databases. Finally, we further predicted the level of PTPRO and the sensitivity of LUAD to multiple chemotherapeutic agents and immunotherapy.

## 2. Methods and Materials

### 2.1. TIMER2.0

Tumor Immune Estimation Resource 2.0 (TIMER2.0) is a public database that can be used to analyze immune cell infiltration in variety of cancers. The database has a variety of analysis modules, such as gene, survival, and copy number variation, to analyze tumor immune function [17].

### 2.2. Acquisition of LUAD Transcription Data from the TCGA Database

The LUAD transcription data was obtained from the TCGA database. The LUAD cohort contains LUAD and normal tissues, and all LUAD tissues contain relevant clinical information. We use corresponding functions in the limma package of the R software to further process these obtained data.

### 2.3. Analysis of Immune Cell Infiltration in LUAD

To investigate the relationship between PTPRO and cellular immune infiltration using multiple databases, including TIMER2.0, TISIDB, and cell type identification by estimating relative subsets of RNA transcripts (CIBERSORT). TIMER and TISIDB are web databases [17, 18]. CIBERSORT identifies immune cell types based on the expression profiles of characteristic genes in RNA-sequencing data. CIBERSORT relies on a gene expression matrix file (named LM22) to parse immune cells in tissues to distinguish human hematopoietic cell phenotypes [19].

### 2.4. Prediction of PTPRO Expression on the Effect of Immunotherapy

The immunophenotype score (IPS) is a predictor of response to anti-CTLA-4 and anti-PD-1 therapy that quantifies determinants of tumor immunogenicity [20]. The principle for this protocol is based on immune-related genes including MHC-associated molecules, checkpoints or immunomodulators, effector cells, and suppressor cells. This method obtains the final IPS by quantifying the abovementioned genes and then weighting them equally.

### 2.5. Prediction of PTPRO Expression on the Efficacy of Chemotherapy Drugs

The principle of this algorithm is based on differentially expressed genes (DEGs) between samples with high and low expressions of the target gene. Then, the top 1000 DEGs will be submitted to the CMap database to match the corresponding potential chemical drugs.

### 2.6. KEGG and GO Enrichment Analyses

GO (http://geneontology.org) and KEGG (https://www.genome.jp/kegg) analyses were frequently used in functional enrichment study and investigated the biological pathways that involve differentially expressed mRNAs. ClusterProfiler (v3.12.0) and Database for Annotation, Visualization and Integrated Discovery tools were conducted to analyze the functional enrichment conditions for dysregulated mRNAs. The false discovery rate (FDR) was calculated to correct the *p* value.

### 2.7. Statistics

In this study, R software was used for calculation and statistical analysis. Their responses to immunotherapy were compared using the Wilcoxon rank-sum test. Differences between high and low target gene classes were determined using Kaplan-Meier curves and log-rank tests. *p* < 0.05 were considered statistically significant.

## 3. Results

### 3.1. PTPRO Is Lowly Expressed in LUAD and Associated with Prognosis

**Figure 1:**
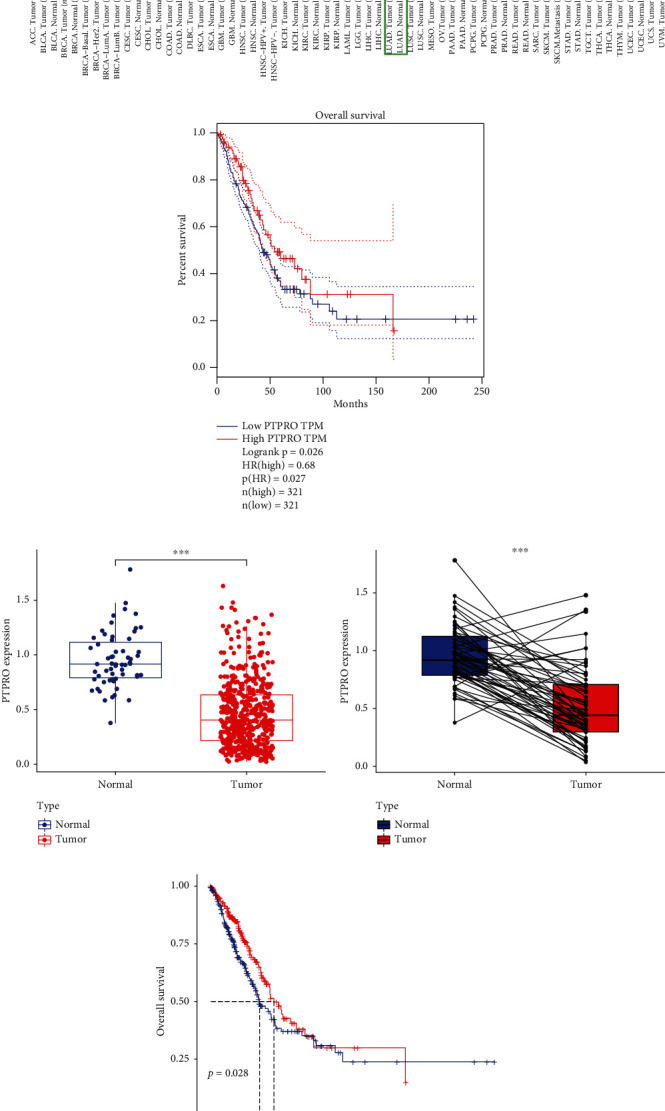
Expression level of PTPRO in LUAD and its correlation with prognosis. (a) Analysis of the expression level of PTPRO in pan-cancer by GEPIA database. (b) Kaplan-Meier survival analysis for high vs. low expression level of PTPRO in LUAD (GEPIA database). (c) Differences in mRNA expression levels of PTPRO in LUAD and normal tissues (TCGA database). (d) Differences in mRNA expression levels of PTPRO in LUAD and paired normal tissues (TCGA database). (e) Kaplan-Meier survival analysis for high vs. low expression level of PTPRO in LUAD (TCGA database).

We first analyzed the expression levels of PTPRO in pan-cancer tissues via the TIMER database. As shown in Figure 1(a), the expression level of PTPRO in LUAD was significantly lower than that in normal tissues. Further survival prognostic analysis showed that LUAD with high PTPRO expression had better prognosis (Figure 1(b)). Furthermore, we analyzed the expression of PTPRO in LUAD by the TCGA database. The results indicated that the expression of PTPRO was significantly higher in LUAD than in normal tissues (Figure 1(c)). The expression levels of PTPRO in LUAD and its paired normal tissues also showed the same results as above (Figure 1(d)). Interestingly, the further analysis revealed that the TCGA-LUAD cohort with a high expression of PTPRO had a better prognosis (Figure 1(e)). These above results indicated that the expression of PTPRO was significantly downregulated and correlated with the prognosis of patients in LUAD.

### 3.2. The Correlation Analysis between the Clinical Features and PTPRO Expression Level for LUAD Patients

**Figure 2:**
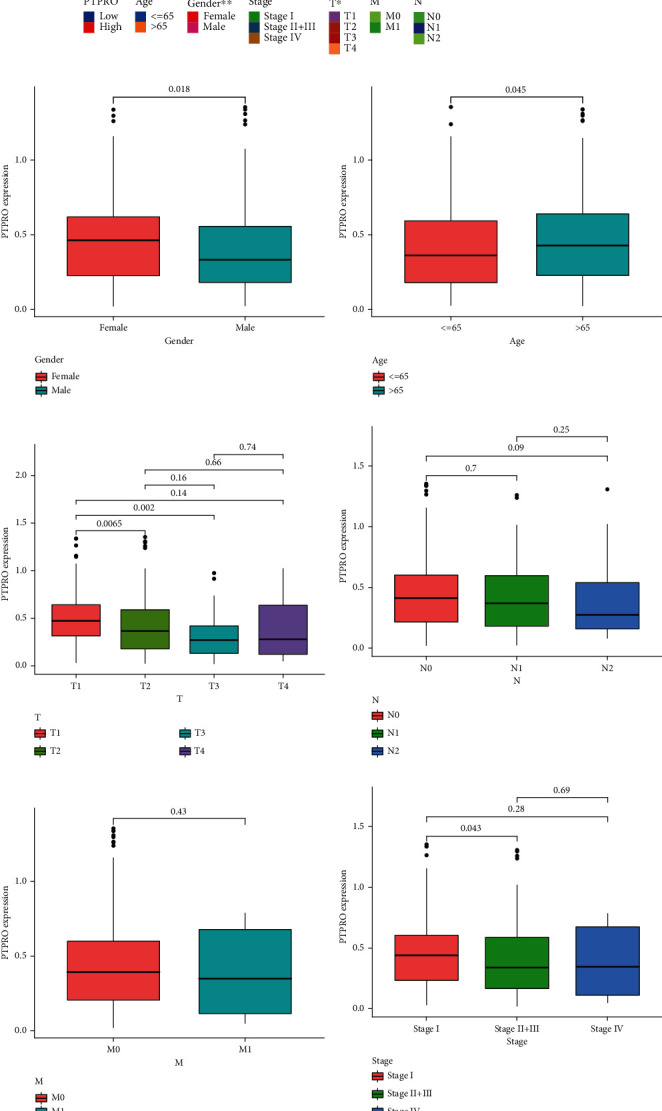
Correlation between the expression level of PTPRO and clinical features in LUAD. (a) Heatmap of the clinical relevance of PTPRO expression level. (b–g) Differences in PTPRO expression level between different clinical subgroups (gender, age, TNM and stage).

The TNM system is widely used in evaluating the classification of LUAD. [21]. Our previous findings suggested that the expression level of PTPRO was closely related to the prognosis of LUAD patients. To further explore the role of PTPRO in LUAD, we analyzed the relationship between PTPRO and clinical characteristics. First, we created a heatmap to show the distribution of clinicopathological feature subtypes in patients with high and low PTPRO expressions (Figure 2(a)). Moreover, we found that PTPRO was expressed at higher levels in female patients, while patients younger than 65 years old had lower levels of PTPRO expression (Figures 2(b) and 2(c)). More interestingly, we found that PTPRO levels were significantly lower in T2 and T3 patients compared to T1 patients (Figure 2(d)). The expression levels of PTPRO in patients were not significantly different in N and M stages (Figures 2(e) and 2(f)). Finally, the correlation between pathological stage and PTPRO level showed that stage II+III patients had lower PTPRO levels than stage I patients (Figure 2(g)).

### 3.3. Construction of a Nomogram Based on PTPRO Expression Levels

**Figure 3:**
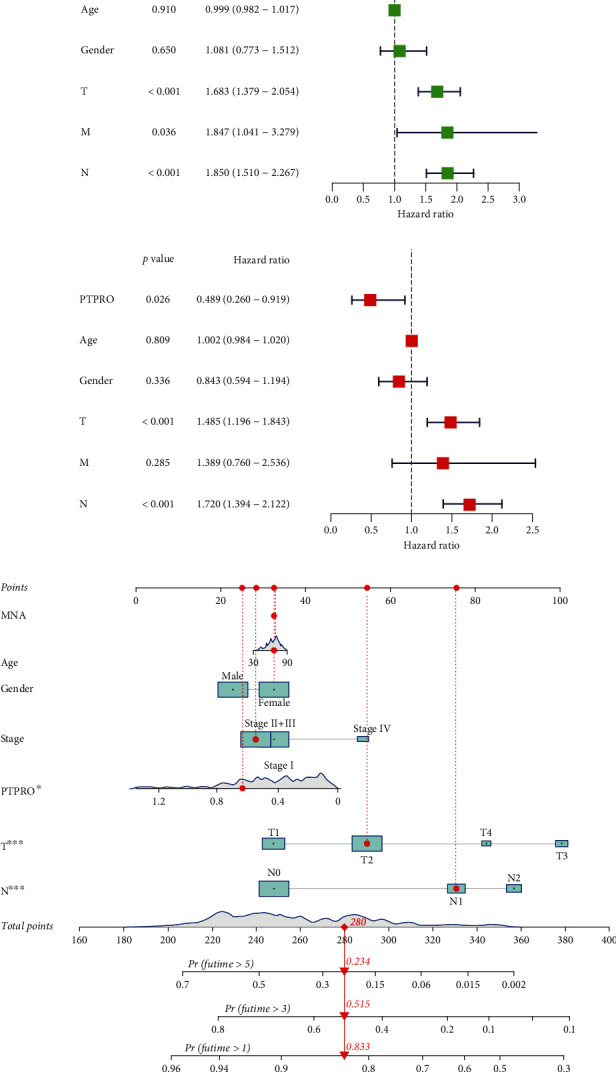
The value of PTPRO expression level in evaluating the prognosis of LUAD. (a, b) Univariate and multivariate regression analyses of PTPRO expression levels and clinical parameters. (c) Construction of nomogram by the PTPRO expression level and clinical characteristics for predicting the probability of 1-, 3-, and 5-year OS of LUAD patients.

The previous results of this study showed that the expression level of PTPRO was positively correlated with the prognosis of various solid tumor and was also closely related to the clinical characteristics [12, 22]. Therefore, we further explored whether PTPRO could be used to assess the prognosis of LUAD. Univariate and multivariate regression analyses indicated that PTPRO was a protective factor for the prognosis of LUAD (Figures 3(a) and 3(b)). Next, we established a nomogram based on PTPRO expression levels and clinicopathological features to predict the prognosis of LUAD (Figure 3(c)). The calibration curve implied that the nomogram has good predictive capacity (Supplementary Figure 1).

### 3.4. Construction of PTPRO-Related Gene Network and Enrichment Analysis of GO and KEGG

**Figure 4:**
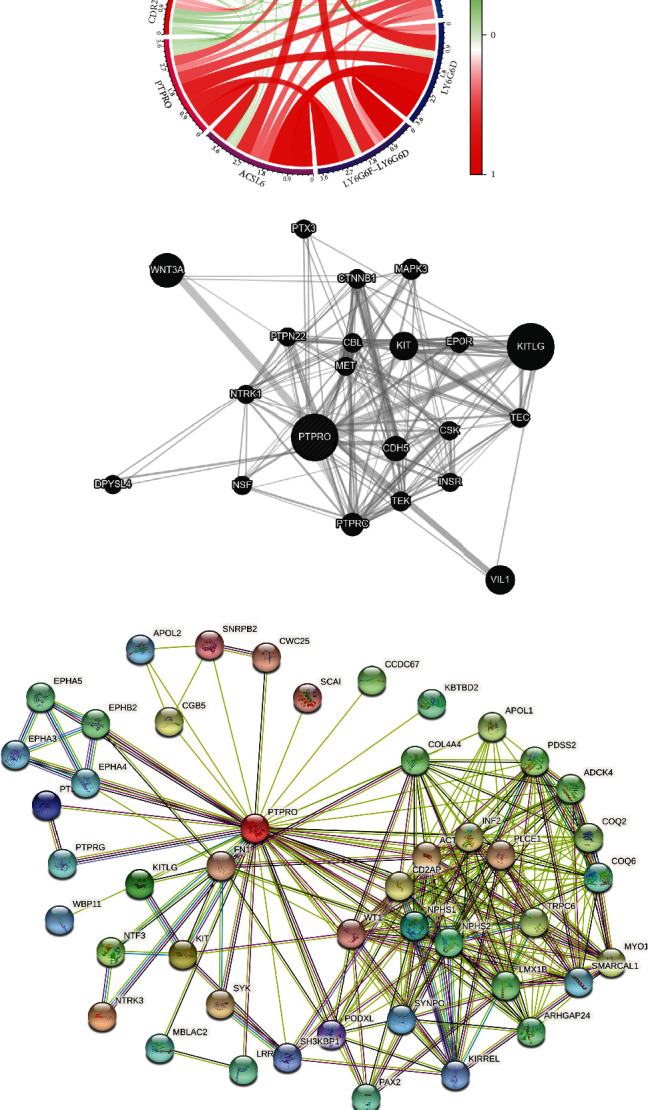
Coexpression network analysis of PTPRO-related genes. (a) Coexpressed genes of PTPRO were analyzed by TCGA-LUAD cohorts. (b) Coexpressed genes of PTPRO were analyzed by GeneMANIA database. (c) Coexpressed genes of PTPRO were analyzed by STRING database.

**Figure 5:**
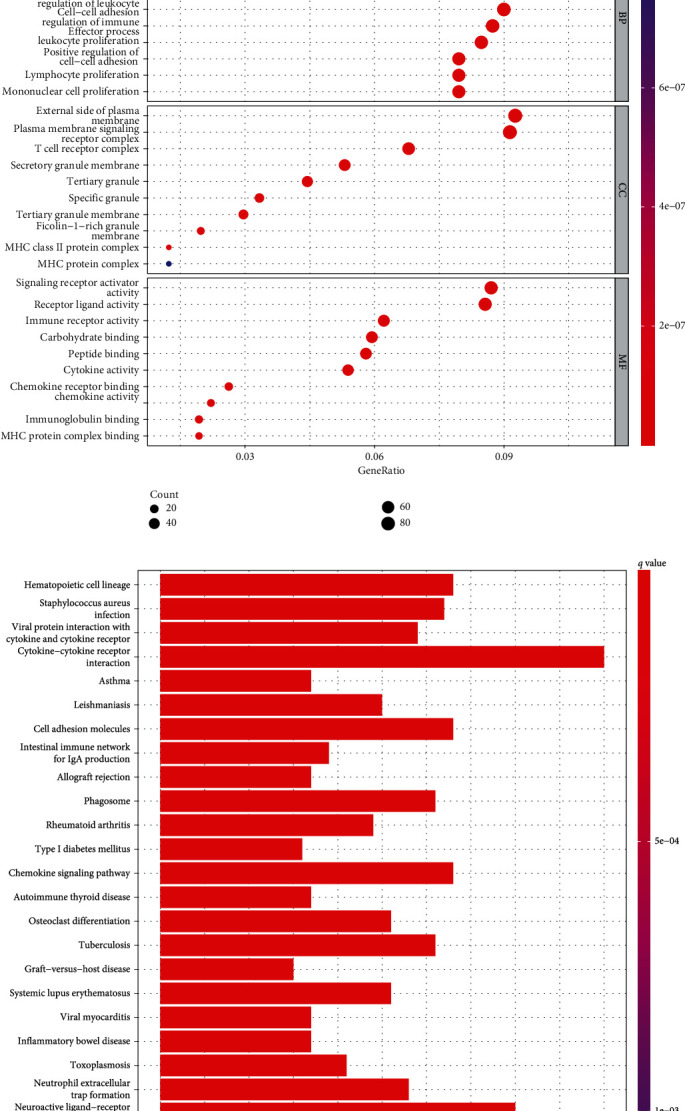
GO and KEGG pathway enrichment analyses for PTPRO-related genes. (a) Heatmap of differential genes (DEGs) with high and low PTPRO expression level in LUAD patients. (b, c) Top 10 results of the GO enrichment analysis based on PTPRO-related DEGs. (d, e) The plots of the KEGG pathway enrichment analysis for PTPRO-related DEGs.

Based on the above results, we constructed PTPRO-related gene networks by multiple databases (TCGA, STRING, and GeneMANIA) to investigate the potential role of PTPRO in LUAD (Figures 4(a)–4(c)). Next, we extracted different expression genes (DEGs) from patients with high and low PTPRO expressions (Figure 5(a)). Furthermore, we performed GO enrichment analysis to clarify the biological processes, cellular components, and molecular function of PTPRO based on the above DEGs. As shown in Figures 5(b) and 5(c), the results showed that the foremost biological processes were leukocyte cell-cell adhesion, T cell activation, and regulation of leukocyte cell-cell adhesion; the top three cellular components were T cell receptor complex, plasma membrane signaling receptor complex, and external side of plasma membrane; the top three molecular functions were immune receptor activity, signaling receptor activator activity, and receptor ligand activity. KEGG enrichment analysis showed that PTPRO was involved in multiple immune-related pathways, including cytokine-cytokine receptor interaction, cell adhesion molecules, chemokine signaling pathway, Th1 and Th2 cell differentiation, and Th17 cell differentiation. These results strongly suggested that PTPRO may be involved in regulating the TIM of LUAD (Figures 5(d) and 5(e)).

### 3.5. Correlation Analysis of PTPRO Expression Level and Immune Cell Infiltration

**Figure 6:**
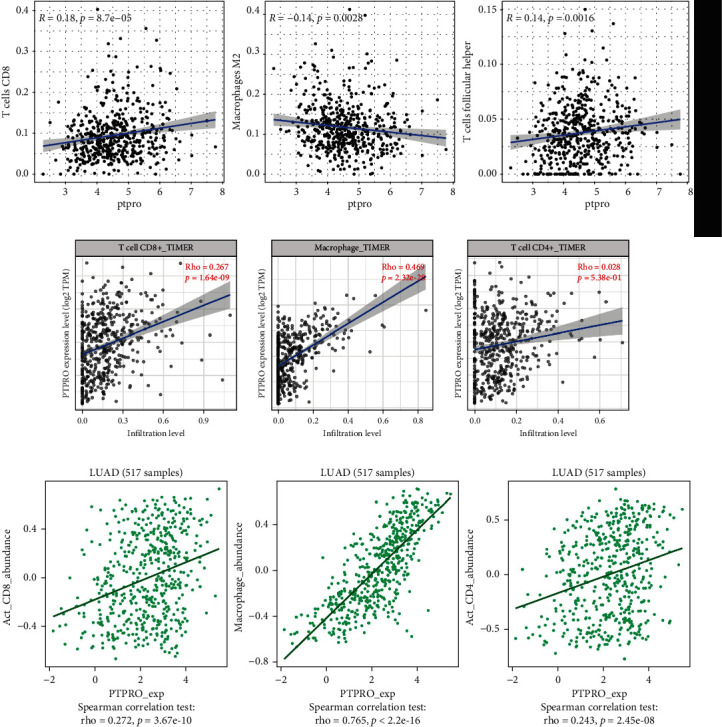
Correlation analysis of PTPRO expression level and immune cell infiltration. (a–c) TIMER, TISIDB, and CIBERSORT tools were used to analyze the correlation between the expression level of PTPRO and the infiltration of LUAD immune cells, respectively.

It is well known that tumor immune dysfunction is a critical step in tumorigenesis and progression [23]. We further analyzed the correlation between PTPRO expression levels and immune cell infiltration via multiple databases. We first analyzed the TCGA-LUAD cohort by the CIBERSORT algorithm, and the results showed that PTPRO was positively associated with a variety of immune cells, including CD8, M2 macrophages, and follicular helper T cells (Figure 6(a)). In addition, the results obtained from the TIMER and TISIDB databases demonstrated that the PTPRO was positively correlated with CD8, CD4 and macrophages (Figures 6(b) and 6(c)). These results indicated that PTPRO may be involved in regulating the infiltration of various immune cells in LUAD.

### 3.6. Prediction of PTPRO Expression Levels within LUAD for Sensitivity to Chemotherapy and Immunotherapy

**Figure 7:**
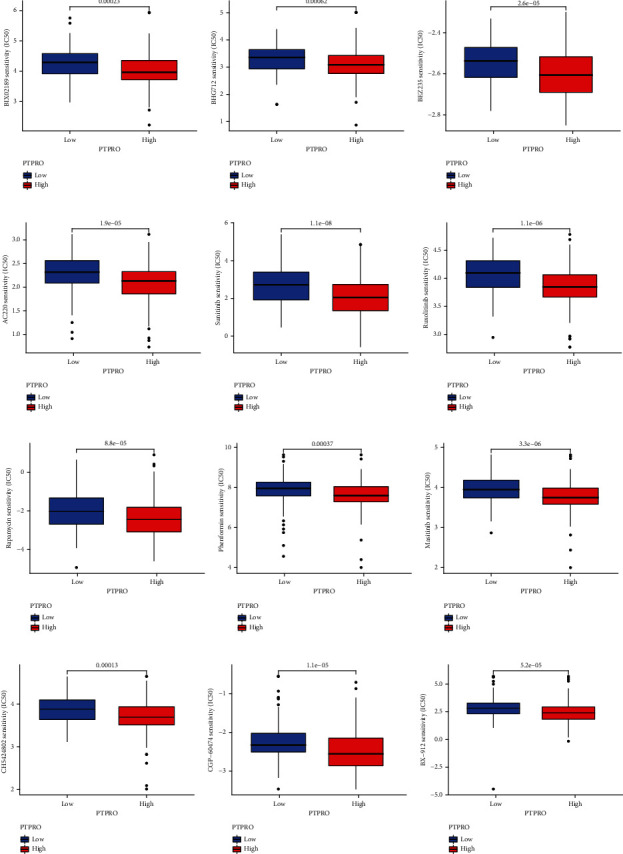
Prediction of sensitivity to chemotherapeutic drugs based on PTPRO expression levels. (a–l) Prediction of PTPRO expression levels in LUAD for sensitivity to multiple chemotherapeutics.

**Figure 8:**
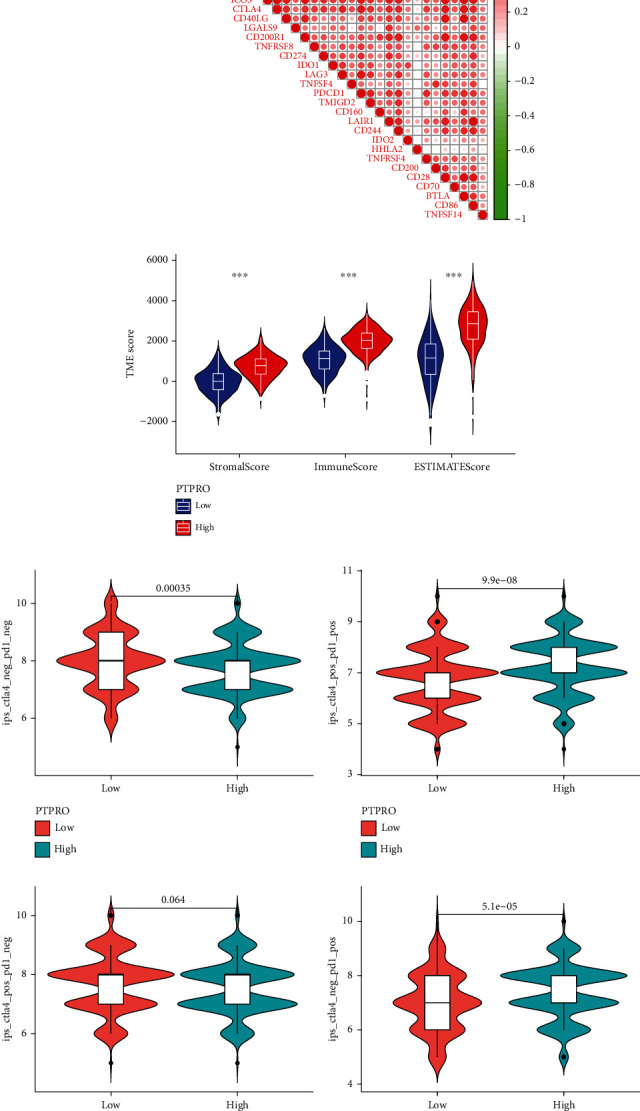
Prediction of sensitivity to immunotherapy based on PTPRO expression levels. (a) Correlation analysis of immune checkpoints and PTPRO expression level. (b) Immune, stromal, and ESTIMATE scores for high and low PTPRO expressions in LUAD. (c–f) The IPS scores between high and low expressions of the PTPRO groups when CTLA-4 or/and PD1 positive.

We analyzed the association between the PTPRO expression level and the chemosensitivity in the treatment of LUAD. As shown in Figure 7, we found that patients with high PTPRO expression showed higher sensitivity to various chemotherapy drugs, such as BLX02189, BHG712, BEZ235, AC220, sunitinib, ruxolitinib, rapamycin, phenformin, masitinib, CH5424802, CGP-06474, and BX912. Given that the function of immune cells is regulated by a variety of immune checkpoints [24]. Therefore, we further analyzed the relationship between PTPRO and various immune checkpoints, and the results demonstrated that the expression level of PTPRO was closely related to various immune checkpoints (Figure 8(a)). Moreover, we evaluated the TIM of LUAD by the ESTIMATE algorithm and observed that LUAD patients with high PTPRO expression had higher TIM scores (Figure 8(b)). Currently, the immunophenoscore (IPS) is a widely used algorithm to predict the immune response [25]. We divided all patients into 4 groups according to the expression of PD1 and CTLA4, namely, PD1_negative_CTLA4_negative, PD1_positive_CTLA4_positive, PD1_negative_CTLA4_positive, and PD1_positive_CTLA4_negative. The results showed that in the PD1_positive_CTLA4_positive and PD1_positive_CTLA4_negative groups, patients with high PTPRO expression had higher IPS scores. In the PD1_negative_CTLA4_negative group, patients with low expression of PTPRO had higher IPS scores, while in the PD1_negative_CTLA4_positive group, the expression level of PTPRO had no effect on the IPS score (Figures 8(c)–8(f)).

## 4. Discussions

LUAD is a highly lethal malignant tumor that seriously threatens the health of human [26]. Conventional treatments, such as surgery, chemotherapy, and radiotherapy, have significantly improved patient outcomes, but further improvements are more difficult [27]. In recent years, immunotherapy for LUAD based on immune checkpoint inhibitors has gradually attracted people's attention [28]. However, only a minority of patients benefit from it, which greatly limits the application of ICIs [5]. In the present study, we found that PTPRO was significantly downregulated in LUAD and positively correlated with patient prognosis. Next, we found that age, gender, T, and stage of patients affected PTPRO expression levels. In addition, we further analyzed the results and showed that PTPRO was a protective factor for LUAD; we further established a nomogram based on PTPRO expression, which was shown to have good predictive capacity for patient prognosis. Furthermore, we preliminarily explored the mechanism of PTPRO in LUAD by GO and KEGG analyses. Moreover, we also found that the expression level of PTPRO was closely related to the infiltration of various immune cells. Finally, we applied an algorithm to predict the sensitivity of PTPRO to chemotherapeutic drugs and immunotherapy response.

Tumor immunotherapy is an extremely complex process, and the execution of leukocyte function is the key to the whole step. The entire tumor immune process mainly includes the following steps: recognition of tumor antigens, presentation of tumor antigens, activation of T cell function, overcoming immune suppression, and killing tumor cells [29–31]. The above steps are not independent but intersect each other. In this study, we confirmed the relationship between PTPRO and the tumor immune microenvironment for the first time, which provided more theoretical support for guiding the immunotherapy of LUAD.

In recent years, a large number of studies have attempted to establish prognostic models based on various gene expressions, in order to provide help for the prognosis of malignant tumors. Guo et al. constructed a prognostic model by analyzing the expression levels of ferroptosis-related lncRNAs from head and neck squamous carcinoma in public databases and confirmed that the model has a good predictive ability for patient prognosis [32]. In the present study, we found that PTPRO was a protective factor for LUAD by univariate and multivariate analyses. Next, we constructed a nomogram based on PTPRO expression levels and multiple clinical characteristics, and the associated calibration curve showed that the nomogram had a good predictive ability for 1-, 3-, and 5-year survival, which indirectly confirmed PTPRO plays a vital role in LUAD.

In this study, we evaluated the relationship between the expression level of PTPRO in LUAD and immune cell subsets in the tumor through multiple databases, and the results showed that the expression level of PTPRO significantly affected the infiltration of various immune cells. It is well known that CD8^+^ cells and macrophages are key cell subsets that perform leukocyte immune function against tumors [31, 33]. A previous study demonstrated that PTPRO can improve TIM in renal cancer, and given the findings we obtained, PTPRO has a similar effect in LUAD [15]. It is worth noting that the results obtained in this study are derived from multiple databases and have high reliability.

Our study demonstrates the important role of PTPRO in LUAD; however, there are still many shortcomings. First, all data in this study were derived from public databases and have not been verified by relevant experiments. In addition, the specific mechanism by which PTPRO regulates the TIM of LUAD has not been fully elucidated in this study.

In conclusion, our results suggested PTPRO expression level is closely related to the prognosis and TIM of LUAD. In LUAD, PTPRO is not only an independent prognostic predictor but also a potential immunotherapy target.

## Data Availability

All data generated in this study are available from the corresponding author.

## Supplementary Materials

Supplementary Materials Supplementary Figure 1: calibration curves of Norman plots constructed based on PTPRO and clinical feature information.
