# Supplementary figures and images for: Comprehensively Analyze the Prognosis Significance and Immune Implication of PTPRO in Lung Adenocarcinoma
Source: Mediators Inflamm. 2023 Feb 9;2023:5248897. doi: 10.1155/2023/5248897 (PMC9934981; doi:10.1155/2023/5248897)

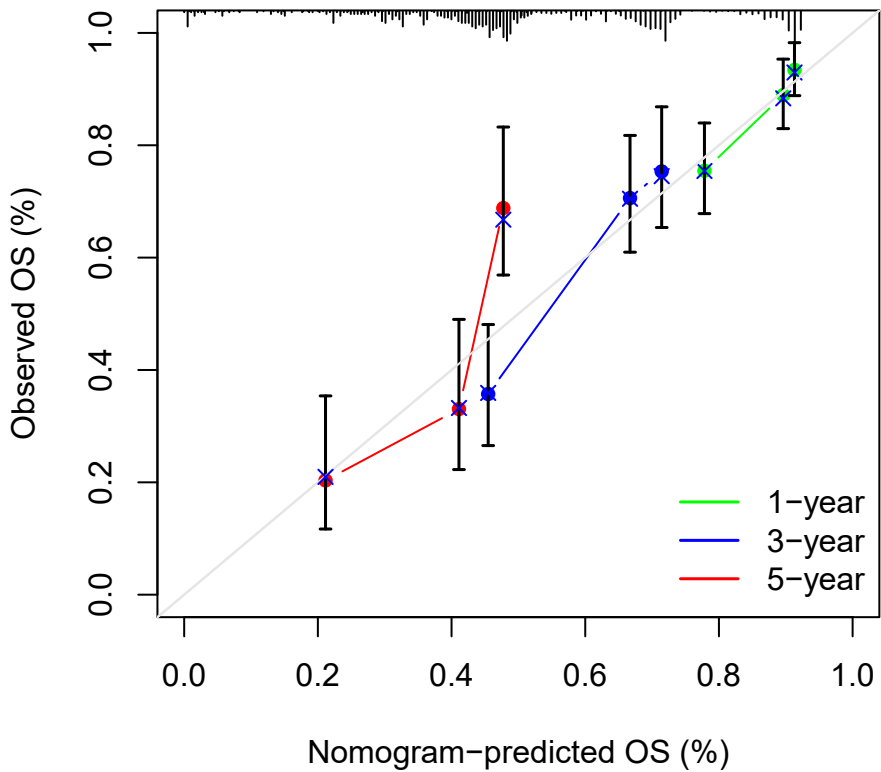

Supplement: Supplementary Materials — Supplementary Figure 1: calibration curves of Norman plots constructed based on PTPRO and clinical feature information. [file 5248897.f1.pdf]
